# Toward a Molecular Classification of Colorectal Cancer: The Role of MGMT

**DOI:** 10.3389/fonc.2013.00266

**Published:** 2013-10-18

**Authors:** Parham Minoo

**Affiliations:** ^1^Calgary Laboratory Services, Department of Pathology, University of Calgary, Calgary, AB, Canada

**Keywords:** MGMT, MSI-H, MSS, MSI-low, CIMP, methylation, DNA repair

## Abstract

O^6^-methylguanine DNA methyltransferase (MGMT) is a DNA repair enzyme with the ability to protect cells from DNA mutations by removing alkyl groups from the O^6^ position of guanine. Colon mucosa is exposed to the direct effects of environmental carcinogens and therefore maintaining a proficient DNA repair system is very important to stay protected against DNA mutagenesis. Loss of MGMT expression is almost exclusively associated with methylation of CpG islands in the *MGMT* gene promoter region which is found in approximately 40% of colorectal cancers. The role of MGMT loss in colorectal tumorigenesis is complex but numerous studies have documented methylation of this gene even in the normal appearing mucosa as well as in aberrant crypt foci, suggesting that MGMT methylation can be regarded as an early event or “field defect” in colon cancer neoplasia. The focus of this perspective is the role of MGMT in different pathways of colorectal carcinogenesis as well as the implication of this molecule in treatment decisions in colorectal cancer patients.

## Introduction

O^6^-methylguanine DNA methyltransferase (MGMT) is a ubiquitously expressed DNA repair enzyme with a unique ability to directly remove alkyl groups from the O^6^ position of guanine. O^6^-alkylguanine adducts cause damage by mispairing with thymine during replication leading to G:C to A:T transitions (1). Therefore MGMT protects normal cells from exogenous carcinogens. For example it has been shown that MGMT protects body against N-nitroso compounds, known to induce colon cancer by methylating the DNA (2). The downside is that MGMT with the same mechanism can protect cancer cells from alkylating chemotherapeutic agents. Each MGMT molecule can only engage in one enzymatic reaction since the active site of MGMT cannot be regenerated. Therefore, upon performing its enzymatic reaction, MGMT is targeted for ubiquitination and degradation (1). Because of this “suicide” mechanism, a cell will have only limited resources to repair abnormal adducts depending on the available numbers of MGMT molecules and the rate of MGMT synthesis. This concept raised many efforts to find an inhibitor for MGMT to be used in clinical practice to overcome resistance to alkylating chemotherapy; however, none of the inhibitors that have been identified showed a clinical advantage in different clinical trials (3). This is partly because of the exacerbation of the toxic side effects of the alkylating drugs due to inactivation of MGMT in normal tissues.

MGMT protein is encoded by *MGMT* gene located at chromosome locus 10q26 (4). The *MGMT* gene has a CpG island containing promoter and thus its expression is significantly regulated by DNA methylation which leads to epigenetic silencing of the gene and loss of MGMT protein expression (1). The most reliable method to evaluate MGMT methylation is a matter of controversy. Methylation specific polymerase chain reaction (MSP) is the most widely used technique with relatively high sensitivity and specificity (5). However, the reliability of MSP is dependent on good quality DNA, which is not typically obtainable from formalin-fixed, paraffin-embedded (FFPE) specimens (6). On the other hand, MSP fails to provide quantitative measurements on MGMT methylation. These limitations constrain the implication of MSP in the clinical setting. Pyrosequencing, combined bisulfite restriction analysis (COBRA), MethyLight, Methylation Sensitive – High Resolution Melting (MS-HRM), Methylation specific multiplex ligation-dependent probe amplification (MS-MLPA) are other semiquantitative or quantitative methods that have been used to evaluate MGMT promoter methylation (7, 8). A recent study investigating the association between MGMT methylation and protein expression showed that MGMT protein expression assessed by immunohistochemistry (IHC) did not correlate with methylation status of MGMT (assessed by MSP) suggesting that MSP and IHC should not be used interchangeably (9).

There are 97 CpG sites present on the promoter region of *MGMT*. Interestingly, these CpG sites do not equally contribute to gene silencing as it has been shown that methylation among these sites is not uniform. Extensive studies have been conducted to map the specific CpG sites that can best predict gene silencing. In one the recent studies, Everhard et al. found six isolated CpG sites (CpGs −228, −186, +95, +113, +135, and +137) as well as two CpG regions (−186 to −172, and +93 to +153), each with a minimum of 81.5% of concordant results between methylation and expression (10). Furthermore, an association between MGMT methylation and the germline C to T SNP (rs16906252) within the first exon of *MGMT* is observed in colorectal cancer and normal colonic mucosa (11, 12).

The impact of MGMT loss in carcinogenesis was first reported in 1999 by Esteller et al. (5). Loss of MGMT expression due to aberrant promoter methylation was shown in 40% of colorectal cancers and gliomas and 25% of non-small cell lung carcinomas, lymphomas, and head and neck carcinomas (5). One year later, the same group documented a link between loss of MGMT and G to A mutations in *K-ras* gene in colon cancer (13), which was followed by a report showing the similar findings in gastric cancers (14). Two other groups described an association between loss of MGMT and G to A mutations in *p53* gene in astrocytomas and non-small cell lung cancers (15, 16). The link between MGMT loss and G to A mutagenesis has been confirmed in subsequent studies (17–19). However, the results of other studies did not support this sequence of changes (12, 20, 21).

## MGMT and Colon Cancer

The role of MGMT loss in colorectal tumorigenesis is complex and not well characterized. *MGMT* methylation has been detected in the aberrant crypt foci, which are the earliest precursor lesions in colon cancer development (22) suggesting that *MGMT* methylation is an early event in neoplastic pathway. Furthermore, low level methylation of *MGMT* has been reported in normal appearing colon mucosa in patients with a correspondingly *MGMT* methylated tumor, as well as individuals without colon cancer (12, 18, 23–25). This finding is suggestive of a role for *MGMT* methylation as a “field defect” in sporadic colon cancer carcinogenesis which is defined as an area of molecularly abnormal tissue that precedes and predisposes to the development of cancer (18). Therefore, it has been proposed that *MGMT* status might be a useful marker for early detection and risk assessment in sporadic colon cancers.

Two major pathways have been described in sporadic colorectal cancers: the chromosomal instability (CIN) pathway and CpG Island Methylator Phenotype (CIMP) pathway. The strong association of MGMT loss with CpG methylation links MGMT to the CIMP pathway, which is associated with BRAF-V600E mutation and MSI-high status (26, 27). In fact *MGMT* methylation has been documented in their precursor lesions, sessile serrated adenoma/polyp (SSA/SSP) (28–31). It has been shown that serrated adenomas with dysplasia are more associated with MGMT methylation compared to hyperplastic polyps and serrated adenomas without dysplasia (31). A recent study reported *MGMT* methylation in 46.7% of microvesicular hyperplastic polyps (MVHP), 60% of SSA/SSP without dysplasia, and 75% of SSA/SSP with dysplasia (32). In supporting of the contribution of MGMT protein in MSI-H pathway of CRC neoplasia, Svrcek et al. reported that field defects resulted from MGMT loss are more frequently associated with MSI-H than microsatellite-stable (MSS) colorectal cancers and concluded that methylation tolerance may represent a crucial initiating step prior to MMR deficiency in the development of MSI-H CRC (24).

On the other hand, the association of *MGMT* loss with G to A transition in *K-ras* and *p53* mutated genes, links MGMT to the CIN pathway of colorectal cancers which is characteristically MSS or -low (MSI-L) and CIMP-low (17, 33–35). The association of MGMT with K-ras in the context of MSS/MSI-L CRCs are not straight forward. For example, a recent study on 776 CRCs revealed that K-ras mutated carcinomas that are associated with *MGMT* methylation, more frequently develop in contiguity with a residual polyp and are associated with different MSI status (36). Jass has suggested a “fusion pathway” with overlapping features from the two major colorectal cancer pathways in which MGMT serves as a “cross-over” point (37). He hypothesized that the “fusion” of the hyperproliferation and crypt fission that characterize adenomas with the inhibition of apoptosis that has been linked with serrated polyps may generate lesions with enhanced aggressiveness. The presence of p53 mutation (likely associated with *MGMT* methylation) in some of the serrated polyps with dysplasia provides an example of this link (37). Another possible link between these two pathways is villous adenoma which, on one hand, is thought to represent an advance lesion in CIN pathway and is frequently associated with K-ras mutation (38). On the other hand, this lesion has morphologic resemblance to the traditional serrated adenoma (TSA) and also harbors K-ras mutation in a subset of cases, likely in association with MGMT methylation (35, 37, 39). Therefore, it has been suggested that villous adenoma may represent a bridge between the two pathways. Despite evidence for involvement of MGMT in colon cancer carcinogenesis, previous studies fail to show any prognostic significance of MGMT methylation (or loss of MGMT) in colorectal cancers (33, 40, 41).

## MGMT in Treatment of Colorectal Cancers

The role of MGMT in response to alkylating chemotherapeutic agents is well studied in glioma patients treated with temazolamide (42, 43). Based on these studies, it is well established that the patients with promoter methylation and loss of MGMT expression have much better response to chemotherapy and also longer progression free and overall survival while the intact expression of MGMT is predictive of a poor response to treatment and worse overall survival (7, 44, 45). As it discussed earlier (see above) this effect is most likely due to the protective function of MGMT against alkylating agents in cancer cells. The significance of MGMT expression in colorectal cancers is less investigated. One of the early studies revealed that CRC patients with unmethylated MGMT promoters who had been treated with chemotherapy were found to have a 5.3-fold greater risk of recurrence than those who had no exposure to chemotherapy (46). The exact mechanism for this finding is not understood as 5FU is an antimetabolite and does not function through alkylation of DNA. Regardless, this finding suggests that CRC patients with intact MGMT expression are not good candidates for 5FU adjuvant chemotherapy. Prior clinical studies did not show a benefit for using alkylating agents in treatment of colorectal cancer. However, given the effect of MGMT loss in sensitizing cancer cells to alkylating agents, recently several attempts were made to select suitable patients for these medications. In a phase II clinical trial study with dacarbazine in metastatic CRC patients who had failed standard therapies, objective clinical response was limited to those patients with *MGMT* methylation (47). Similar findings were seen in metastatic patients with MGMT methylation who were treated with single agent Temozolomide (48). This data opens a new window for an effective treatment in patients with colon cancer who are deficient in MGMT and represent an example of a personalized approach in treatment of cancers.

## Conclusion

Colorectal cancer is a heterogeneous disease arising in association with abnormalities in different molecular pathways. The fine dissection of molecular events is necessary to establish molecular signatures that can correctly classify CRCs and reliably predict tumor behavior and prognosis. This article is a part of an attempt to put together our current knowledge about molecular mechanisms in CRC under the title of “Toward molecular classification of colorectal cancer.” The role of MGMT protein in colorectal carcinogenesis is rather complex and poorly understood. However, based on the available data there are grounds to believe that MGMT plays an important role in development of CRC and may represent a bridge between different molecular pathways. Further studies are required to shed light on the contribution of this molecule in colorectal neoplasia.

## Conflict of Interest Statement

The author declares that the research was conducted in the absence of any commercial or financial relationships that could be construed as a potential conflict of interest.
